# Insomnia and risk of chronic musculoskeletal complaints: longitudinal data from the HUNT study, Norway

**DOI:** 10.1186/s12891-018-2035-5

**Published:** 2018-04-25

**Authors:** B. L. Uhlig, T. Sand, T. I. Nilsen, P. J. Mork, K. Hagen

**Affiliations:** 1 Department of Neuromedicine and Movement Science, Norwegian University of Science and Technology (NTNU), PB 8905, MTFS, N-7489 Trondheim, Norway; 2 Department of Neurology and Clinical Neurophysiology, St. Olavs Hospital, N-7006 Trondheim, Norway; 3 Norwegian Advisory Unit on Headaches, St. Olavs Hospital, N-7006 Trondheim, Norway; 4 Department of Public Health and Nursing, Norwegian University of Science and Technology (NTNU), N-7491 Trondheim, Norway; 5 Clinic of Anaesthesia and Intensive Care, St. Olavs Hospital, N-7006 Trondheim, Norway

**Keywords:** Sleep, Pain, Musculoskeletal pain, Prospective, Epidemiology

## Abstract

**Background:**

The aim of this study was to investigate the prospective association between insomnia and risk of chronic musculoskeletal complaints (CMSC) and chronic widespread musculoskeletal complaints (CWMSC). A second aim was to evaluate the association between insomnia and number of body regions with CMSC at follow-up.

**Methods:**

We used data from the second (HUNT2, 1995–1997) and third (HUNT3, 2006–2008) wave of the Nord-Trøndelag Health Study (the HUNT Study). The population-at-risk included 13,429 people aged 20–70 years who reported no CMSC at baseline in HUNT2 and who answered the questionnaires on insomnia in HUNT2 and CMSC in HUNT3. Insomnia was defined according to the 4th edition of the Diagnostic and Statistical Manual of Mental Disorders (DSM-IV) with minor modification, whereas CMSC was assessed for nine different body regions. CWMSC was defined according to the 1990 criteria by the American College of Rheumatology. We used Poisson regression to estimate adjusted risk ratios (RRs) for CMSC and CWMSC at 11 years follow-up. Precision of the estimates was assessed by a 95% confidence interval (CIs).

**Results:**

Insomnia at baseline was associated with increased risk of any CMSC (RR 1.16, 95% CI 1.03–1.32) and CWMSC (RR 1.58, 95% CI 1.26–1.98) at follow-up. RR for CMSC for specific body regions ranged from 1.34 (95% CI 1.05–1.73) for the knees and 1.34 (1.10–1.63) for the neck to 1.60 (95% CI 1.19–2.14) for the ankles/ft. Further, insomnia was associated with increased risk of CMSC in 3–4 regions (RR 1.36, 95% CI 1.05–1.77), and 5 or more regions (RR 1.93, 95% CI 1.40–2.66), but not 1–2 regions (RR 0.99, 95% CI 0.80–1.24).

**Conclusions:**

Insomnia is associated with increased risk of CMSC, CWMSC, and CMSC located in 3 or more body regions.

## Background

Pain, chronic pain and chronic widespread pain are important contributors to years lived with disability and disability-adjusted life years [1–3]. Their high prevalence [4], and the negative impact on individuals as well as on the society, underscore the importance of identifying modifiable risk factors that can serve as target for preventive interventions.

The definitions of chronic musculoskeletal pain and chronic widespread musculoskeletal pain differ widely between studies. However, according to the 1990 criteria of the American College of Rheumatology (ACR) [5], the term “chronic” means presence of symptoms for at least ≥3 months during the last year. Furthermore, “chronic widespread musculoskeletal pain” is defined as pain with axial skeleton pain, pain in the left and the right of the body, and pain above and below the waist [5].

Insomnia is one potentially modifiable risk factor, and an association between sleep problems and musculoskeletal pain has been found in cross-sectional studies [6–8]. Moreover, prospective studies have shown that sleep problems increase the risk of chronic pain in the neck, shoulders and low back [1, 9, 10], as well as chronic widespread pain [11, 12]. Methodological differences, such as study design (i.e. sample size, population-based or selected groups, cross-sectional or prospective), duration of follow-up, and definition of chronic musculoskeletal pain and insomnia make it difficult to compare studies. To clarify the influence of insomnia on the risk of chronic musculoskeletal pain, large-scale population-based studies with standardized disease criteria are warranted. Further, chronic widespread musculoskeletal pain may represent a disorder different in nature from localized pain [13]. Similarly, while not an established hypothesis, the pathophysiology behind chronic pain potentially differs between regions (e.g. axial skeleton versus upper- and lower limbs). Thus, evaluating the association between insomnia and risk of chronic pain in specific body regions may be of interest.

The 1990 criteria of the American College of Rheumatology (ACR) [5] have recently been replaced by the Widespread Pain Index (WPI) in the ACR preliminary 2010 criteria of FM [14]. In the WPI, number of pain sites is counted. With this recent change in the definition of FM in mind, it is relevant to evaluate the prospective association between insomnia and number of chronic musculoskeletal pain regions.

The present study is a large-scale adult population-based cohort study of participants without chronic musculoskeletal complaints (CMSC) at baseline. The term “complaints” was used rather than “pain”, because the screening question included the words “pain and/or stiffness” to an extent that was bothersome.

The primary aim of this study was to investigate the prospective association between insomnia and risk of developing CMSC and chronic widespread musculoskeletal complaints (CWMSC). A second aim was to evaluate the association between insomnia and number of body regions with CMSC at follow-up.

## Methods

### Study design

This is a population-based cohort study that evaluates the prospective association between insomnia at baseline and risk of CMSC and CWMSC at a mean follow-up time of 11 years (range 9–13 years).

### The Nord-Trøndelag Health (HUNT) surveys

All inhabitants of Nord-Trøndelag county in Norway 20 years and older have been invited to three surveys, HUNT1 (1984–1986), HUNT2 (1995–1997), and HUNT3 (2006–2008). In all three surveys, information on lifestyle and health-related factors was collected by questionnaires and a brief medical examination, including measurements of blood pressure, height, and weight. HUNT1 did not include information on musculoskeletal complaints, and the present study is therefore based on data from HUNT2 and HUNT3. More detailed information about the HUNT Study can be retrieved online [15].

### Chronic musculoskeletal complaints

At HUNT2, all participants were asked a CMSC screening question: “During the last year, have you had pain and/or stiffness in your muscles and/or joints that has lasted for at least 3 consecutive months?” (yes, no). In the present study, individuals who responded “no” to the screening question were considered as being without CMSC at baseline in HUNT2. HUNT3 was to a large extent a replication of HUNT2 [16] and included an identical screening question on CMSC. Individuals who answered “yes” to the screening question (pain and/or stiffness in muscles and joints for ≥3 months during the past year) were defined as having CMSC. These were asked to mark the pain location(s), with the following nine options: neck, shoulders, upper back, elbows, lower back, hands/wrists, hips, knees, ankles/ft. Furthermore, based on information about body regions with CMSC, the participants were also categorized into 1–2 body regions, 3–4 body regions, or ≥ 5 body regions. CWMSC were defined according to the 1990 criteria of the American College of Rheumatology (ACR) as CMSC with axial skeleton pain (neck, upper back or lower back), pain in the left and the right of the body, pain above the waist (neck, shoulders, elbows, wrist/hands or upper back), and below the waist (lower back, hips, knees or upper back). Also, having CMSC in both the left and right side of the body was required.

Unfortunately, the “anterior chest” region (as included by ACR) was not included among the different pain locations in HUNT3. Moreover, the participants were not asked specifically for left or right-sided complaints for each separate region, but one general question on whether they had CMSC in both sides of the body.

The reliability of self-reported CMSC and diagnosis of CWMSC has been reported previously, by comparing answers in the questionnaire with those made in a clinical interview [4]: the change-adjusted agreement (kappa value) for CMSC was 0.63, 95% CI 0.53–0.73), and for CWMSC 0.48 (95% CI 0.38–0.64).

### Insomnia classification

Based on three questions asked in HUNT2, a proxy for the insomnia diagnosis according to the 4th edition of the Diagnostic and Statistical Manual of Mental Disorders (DSM-IV) [17] was constructed. The DSM-IV asks for difficulty initiating or maintaining sleep and experiencing non-restorative sleep (i.e. sleep problems at night affected the person also at daytime) for a period of 1 month or more [17, 18]. Two of the questions at HUNT2 concerned difficulty falling asleep and early awakening: “During the last month, have you had difficulty falling asleep?” (never, sometimes, often, almost every night) and “During the last month, have you woken up too early and not fallen asleep again?” (never, sometimes, often, almost every night). One question asked for the impact of sleeplessness on work ability: “During the preceding year, have you been bothered by sleeplessness, to an extent that it affected your work ability?” (yes, no). Only participants age < 70 years were asked this question. The proxy diagnosis required either difficulty falling asleep or early awakening “often” or “almost every night” for the last month, in addition to impaired work ability the preceding year. An identical classification of insomnia has been used in previous studies [18]. Those who had answered at least one of three insomnia questions but did not fulfil these criteria, were considered not to have insomnia.

### Other variables

Participants were also asked questions regarding leisure time physical activity, smoking, education and anxiety and depression measured by the Hospital Anxiety and Depression Score HADS [19]. Details on the phrasing of these questions have been described previously [20–23]. For the purpose of this study, leisure time physical activity was categorized by intensity and duration per week: ≥3 h hard physical activity, 1–2 h hard physical activity, ≥3 h light physical activity, 1–2 h light physical activity and physical inactivity (< 1 h) [24]. Smoking was categorized as daily smoker, former smoker, and never smoked. Education level was categorized according to duration: ≤9 years, 10–12 years, and ≥ 13 years. Body mass index (BMI) was subdivided into three groups: underweight/normal weight (< 25 kg/m^2^), overweight (25–29.9 kg/m^2^), and obese (≥30 kg/m^2^) according to the cut points suggested by the World Health Organization [25]. Age was divided into 5-year categories. The total HADS was dichotomized using a cutoff of < 16 vs. ≥16 [26].

### Study population

A total of 37,071 individuals participated in both HUNT2 and HUNT3 (representing 44.6% of the remaining individuals residing in the county and 39.4% of those invited to HUNT2) [27].

**Fig. 1:**
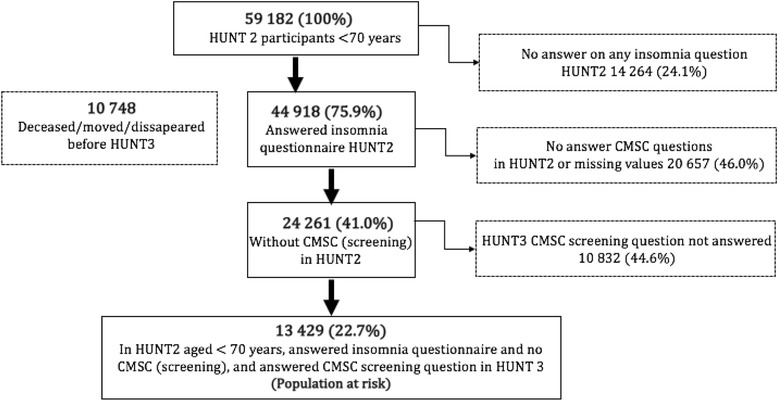
Inclusion of population at risk. Abbreviations: HUNT: Nord-Trøndelag Health Survey; CMSC: Chronic musculoskeletal complaints

In HUNT2, only participants aged < 70 years were asked the question regarding the impact of sleeplessness on working ability. Thus, the population-at-risk in the present study was participants who 1) had no CMSC (screening question), were below 70 years of age and answered ≥1 questions on insomnia in HUNT2 and 2) answered the CMSC screening question in HUNT3. A total of 13,429 individuals aged 20–70 years reported no CMCSs at baseline in HUNT2 and responded to all relevant questions in HUNT2 and HUNT3. A flow chart of participants in HUNT2 and HUNT3 is shown in Fig. 1.

### Statistics

Poisson regression with robust variance estimation [28] were used for to estimate risk ratios (RRs) with 95% confidence intervals (CI) for CMSC and CWMSC at follow-up (HUNT3). To assess the association between insomnia and number of body regions affected by CMSC we constructed three different outcome variables that were analyzed separately; no CMSC vs 1–2 affected regions; no CMSC vs 3–4 affected regions; and no CMSC vs 5 or more affected regions. This approach has been recommended for ordinal outcome data for ease of interpretation [29]. All analyses were initially adjusted for sex and age (5-year categories), and in fully adjusted models we also included leisure time physical activity, smoking, education, HADS, and BMI*.* As the data set allowed for enough cases in each group, categorical variables were used for all continuous variables in case of non-linear relationships. A separate “missing” category for each variable was included in the analyses. Thus, within the population-at-risk, all missing answers on covariates were included in the model. This was done to prevent selection bias, we assumed missing answers was not random. However, 62 who had answered “yes” to the screening question but not marked a specific body region were excluded in analyses on type and number of body regions. Potential effect modification between insomnia and other factors (sex, age, education level, smoking, physical activity, HADS and BMI) was evaluated by including the product of the variables (e.g. insomnia and sex, insomnia and age) in the regression model. Analyses were carried out using IBM SPSS statistics version 21 (Chicago, IL, USA).

## Results

Table 1 presents baseline characteristics of the study population by insomnia status. At baseline, the proportion of participants classified with insomnia was 2.4%. People with insomnia were more likely to be current smokers, had higher HADS score, as well as a higher education level.

**Table 1:** Baseline characteristics of population at risk

	No insomnia	Insomnia
No. of participants	13,113	316
No. of females (%) (missing = 0)	7185 (54.8)	181 (57.3)
Mean age, years (SD) (missing = 0)	43.4 (12.2)	44.5 (12.2)
Education ≥13 years, no (%) (missing = 167)	3680 (28.4)	108 (34.7)
Paid or self-employed workers, no (%) (missing = 113)	9947 (76.5)	211 (67.6)
Heavy physical activity ≥3 h/w^b^, no (%) (missing = 418)	1390 (10.9)	35 (11.6)
Daily smokers, no (%) (missing = 131)	3024 (23.3)	95 (30.4)
HADS^c^ mean, (SD) (missing = 141)	6.2 (4.6)	12.7 (6.8)
Body mass index ≥25 (%) (missing =26)	7181 (54.9)	156 (49.5)

Abbreviations: *SD* standard deviation, *no* number, *h/w* hours per week, *HADS* Hospital Anxiety and Depression Score

### The impact of insomnia on the occurrence of CMSC by location

Table 2 shows the association between insomnia at baseline and risk of any CMSC, CWMSC and region-specific CMSC at follow-up. There was no evidence that the association between insomnia and risk of CMSC was modified by gender (P_interaction_ = 0.34), and thus all results are presented for men and women combined.

**Table 2:** Relative risks (RR) of CMSC in different body regions at 11-year follow-up, according to insomnia status at baseline (316 with insomnia and 13,113 without). Outcome: type of CMSC vs. no CMSC

Type of CMSC	No. of cases (prevalence)	Partly ^a^ adjusted RR (95% CI)	Fully ^b^ adjusted RR (95% CI)
CMSC screening			
No insomnia	4765 (36.3%)	1.00	1.00
Insomnia	144 (45.6%)	1.25 (1.10–1.41)	1.16 (1.03–1.32)
CWMSC			
No insomnia	1338 (10.2%)	1.00	1.00
Insomnia	62 (19.6%)	1.90 (1.53–2.35)	1.58 (1.26–1.98)
Neck			
No insomnia	1950 (14.9%)	1.00	1.00
Insomnia	74 (23.4%)	1.58 (1.31–1.92)	1.34 (1.10–1.63)
Shoulder			
No insomnia	2314 (17.6%)	1.00	1.00
Insomnia	89 (28.2%)	1.57 (1.32–1.86)	1.41 (1.18–1.68)
Upper back			
No insomnia	798 (6.1%)	1.00	1.00
Insomnia	29 (9.2%)	1.69 (1.20–2.38)	1.40 (0.99–1.97)
Elbow			
No insomnia	642 (4.9%)	1.00	1.00
Insomnia	25 (7.9%)	1.80 (1.24–2.60)	1.56 (1.07–2.27)
Lower back			
No insomnia	1822 (13.9%)	1.00	1.00
Insomnia	69 (21.8%)	1.59 (1.30–1.95)	1.36 (1.11–1.68)
Hands/wrist			
No insomnia	1065 (8.1%)	1.00	1.00
Insomnia	38 (12.0%)	1.57 (1.18–2.10)	1.35 (1.01–1.82)
Hips			
No insomnia	1412 (10.8%)	1.00	1.00
Insomnia	60 (19.0%)	1.74 (1.40–2.17)	1.56 (1.24–1.95)
Knees			
No insomnia	1396 (10.6%)	1.00	1.00
Insomnia	50 (15.8%)	1.53 (1.20–1.95)	1.34 (1.05–1.73)
Ankles/Feet			
No insomnia	924 (7.0%)	1.00	1.00
Insomnia	40 (12.7%)	1.83 (1.39–2.42)	1.60 (1.19–2.14)

Abbreviations: *CMSC* chronic musculoskeletal complaints, *CWMSC* chronic widespread musculoskeletal complaints, *CI* confidence interval

^a^ Adjusted for gender and age

^b^ Adjusted for gender, age, education, smoking, physical activity, Hospital Anxiety and Depression Score, Body Mass Index

In the fully adjusted model, individuals with insomnia had increased risk of CMSC (RR 1.16, 95% CI 1.03–1.32) and CWMSC (RR 1.58, 95% CI 1.26–1.98) compared to people without insomnia. Further, insomnia at baseline was associated with increased risk of CMSC in all regions, with RRs ranging from 1.34 (95% CI 1.05–1.73) for the knees and 1.34 (1.10–1.63) for the neck to 1.60 (95% CI 1.19–2.14) for ankles/ft.

A significant interaction was found for education level (P_interaction_ = 0.03). No other factors had interaction with insomnia. Because of the interaction, a supplementary stratified analysis was done: In fully adjusted analyses separated by education level, RRs for CMSC were respectively 1.31 (1.05–1.65) for ≤9 years, 0.96 (0.78–1.19) for 10–12 years, and 1.25 (1.00–1.56) for ≥13 years.

### The impact of insomnia on the occurrence of CMSC by number of affected regions

Table 3 shows the association between baseline insomnia and risk of CMSC in multiple body regions at follow-up. Insomnia was associated with a RR of 1.36 (95% CI 1.05–1.77) for reporting CMSC in 3–4 regions, and a RR of 1.93 (95% CI 1.40–2.66) for reporting CMSC in five or more regions. There was no association between insomnia and reporting CMSC in 1–2 CMSC regions (RR 0.99, 95% CI 0.80–1.24).

**Table 3:** Relative risks (RR) of CMSC categorized by number of body regions at 11-year follow-up, according to insomnia status at baseline (316 with insomnia and 13,113 without). Outcome: Grouped no. of body regions vs. no CMSC

No. of body regions	No. of cases ^a^	Partly ^b^ adjusted RR (95% CI)	Fully ^c^ adjusted RR (95% CI)
1–2			
No insomnia	2768 (21.1%)	1.00	1.00
Insomnia	61 (19.3%)	1.05 (0.85–1.30)	0.99 (0.80–1.24)
3–4			
No insomnia	1403 (10.7%)	1.00	1.00
Insomnia	48 (15.2%)	1.51 (1.17–1.95)	1.36 (1.05–1.77)
5 or more			
No insomnia	581 (4.4%)	1.00	1.00
Insomnia	34 (10.8%)	2.46 (1.81–3.35)	1.93 (1.40–2.66)

Abbreviations: *CMSC* chronic musculoskeletal complaints, *CI* confidence interval

^a^ 62 participants with CMSC did not report body region

^b^ Adjusted for gender and age

^c^ Adjusted for gender, age, education, smoking, physical activity, Hospital Anxiety and Depression Score, Body Mass Index

## Discussion

In this large prospective population-based cohort study, we found that insomnia at baseline was associated with increased risk of CMSC and CWMSC at 11 years follow-up. Furthermore, insomnia increases the risk of CMSC in all body regions.

### Comparison with other studies

Several population-based prospective studies have investigated the association between sleep problems and risk of CMSC or chronic pain [1, 11, 30, 31]. However, the criteria for CMSC or unspecified musculoskeletal pain and insomnia or sleep problems have varied, and the vast majority of other studies had smaller sample sizes.

One previous study from the same population has reported on the association between sleep problems and risk of chronic pain in the neck/shoulders and back [1]. Results were in line with the current study, i.e., sleep problems at baseline increased the risk of chronic pain in the neck/shoulders and low back, and severity of sleep problems seemed to be dose-dependently associated with risk [1]. However, the study used a one general question on sleep problems, and data from HUNT1 and HUNT2 were used in the study. As HUNT1 lacks information on musculoskeletal complaints, baseline CMSC was not assessed.

To our knowledge, no population-based studies have investigated the association between insomnia requiring daytime consequences and risk of CMSC in several body regions. In a population-based study from the UK, baseline insomnia was associated with new-onset of chronic widespread pain at 15-months follow-up [11]. Similar to our study, the definition of chronic widespread pain was based on the 1990 ACR criteria, but a sleep problem scale was used to assess sleep problems. Furthermore, in a study from the general UK population, Morphy et al. [12] found higher risk of widespread pain in those with insomnia at baseline. Although daytime symptoms of insomnia were included in that study, these symptoms were not a requirement in the main analyses. Widespread pain was defined as at least 4 body regions with pain, but pain both in the left and right side of the body was not required. Jansson-Fröjmark et al. [31] found insomnia symptoms to be associated with persistence of pain in a Swedish population, but not increased risk of new-onset pain, which is in contrast with the findings in our study. However, in that study, insomnia symptoms were assessed by a yes/no question regarding sleep problems in the last 3 months (no daytime consequences required). On the other hand, pain was assessed by frequency during the last 12 months, and contrasting our study they also took daytime consequences of pain into account.

### Interpretation

In the present study, we found that insomnia at baseline increased the risk of CMSC in 3–4 or more regions. Correspondingly, a recent study found that CMSC was associated with increased risk of insomnia, most evident among those with CMSC in at least 5 body regions [32]. These two HUNT-based studies provide evidence of bidirectional influence, with each disorder increasing the risk of onset of the other. CMSC and insomnia could be causally related, with insomnia causing CMSC, and vice versa. However, shared underlying mechanisms should also be considered.

Increased pain sensitivity could be one such common predisposition, increasing risk of generalized but not localized pain. Central sensitization has indeed been discussed as a major contributing mechanism in chronic pain syndromes like fibromyalgia (FM) and chronic tension-type headache, and poor sleep has also shown to be correlated to central sensitization (measured by tender point count and algometry (pain sensitivity to pressure)) [33]. Inflammatory mediators, opioid neuropeptide mechanisms or disturbed attentional top-down inhibitory CNS pain modulation [34, 35] are further possible mechanisms in the development of chronic pain in insomniacs. On the other hand, affective mechanisms may explain, at least in part, the association between poor sleep, increased pain sensitivity and CWMSC [36]. In the present study, we performed adjustments for total HADS and the significant associations remained, suggesting that other mechanisms, e.g. a shared genetic predisposition for sleep disturbance and pain are also involved [37].

The insomnia prevalence of 2.4% at baseline in HUNT2 was very low compared to most other population-based insomnia studies [38]. Most likely, two main factors explain this low insomnia prevalence. Firstly, the insomnia prevalence was estimated in the population-at-risk, excluding individuals with CMSC. Secondly, the present criteria of insomnia were more stringent than in most other studies, requiring impact on work ability as a criterion of daytime consequence. A previous published HUNT2 study reported a prevalence of insomnia of 13.5% including all participants (also those with CMSC), and with a less strict insomnia definition, not requiring impaired work ability [6]. Finally, supplementary analyses were performed because of an interaction between insomnia and education level. However, this stratified analysis revealed no consistent differences between RRs on education level. Because interaction for all variables was evaluated, the probability of a random finding is high.

### Strengths

Important strengths of this study include the prospective design, the exclusion of individuals with CMSC at baseline, and the possibility of adjusting for several potential confounding factors with known impact on CMSC and insomnia, such as age, gender, education [39, 40], physical activity [40], BMI [40, 41], smoking [40, 42] and HADS score [40]. The reliability of the questions on CMSC and CWMSC status to a clinical interview was found to be moderate to good [4].

In the present study, insomnia was classified according to the (DSM-IV) [17] with minor modification, and similar criteria have been used in other studies [43, 44]. Including some daytime consequences as in our study is a required criterion in both the DSM-IV [17], the DSM-V [45], the ICD-10 [46], and the ICSD-3 [47]. However, more recent criteria for insomnia have been published since HUNT2 and both DSM-V [45] and ICSD-3 [47] use a 3-month criterion for duration of symptoms. In addition, a question on work ability does not encompass all possible consequences of non-restorative sleep. To our knowledge this is the first study using a proxy diagnosis for insomnia that includes daytime consequences as a requirement, when investigating risk of CMSC and CWMSC.

### Limitations

Some limitations should be considered when interpreting the results. As in other studies diagnosing insomnia with a questionnaire, the lack of objective sleep measures implies that the influence of other sleep disorders (e.g. sleep apnea) on the proxy insomnia diagnosis could result in misclassification. Further, although insomnia has been shown to be a persistent condition [43, 48], changes in insomnia during follow-up could not be taken into account, as we have no information on insomnia during the follow-up period. However, the follow-up period was assumed long enough for potential CMSC caused by insomnia to develop. Furthermore, the possibility for selection bias cannot be fully excluded, although selective participation in HUNT3 based on answers of insomnia question in HUNT2 and status of CMSC in HUNT3 seems unlikely [4]. It should be noted that CMSC includes both pain and/or stiffness in muscles and joints. While not necessarily a limitation, it should be taken into account when comparing results with other studies that have focused exclusively on pain. Because stiffness is generally regarded as less bothersome than pain, there is reason to believe that very few participants who answered the CMSC question were bothered enough by stiffness alone to report it as CMSC. However, differences in the pathophysiology of stiffness and pain should nevertheless be considered in the interpretation of the study, and the threshold of reporting bothersome CMSC may be lower when stiffness is taken into account in addition to pain.

Also, the ACR criteria for widespread pain does not include the term “stiffness”, and also uses “anterior chest” as a region for axial pain, this particular region was not included in our study. Participants were not asked specifically for left or right-sided complaints for each separate location, but one general question on whether they had CMSC in both sides of the body. In the insomnia diagnosis, we included all who answered the insomnia questionnaire. Among those who had answered the insomnia questionnaire (i.e. at least one question), a missing response on one of the remaining questions was assumed to be a negative answer. However, in principle these questions could have been positive, thus artificially reducing insomnia prevalence in HUNT2.

Although we excluded participants without CMSC in HUNT2, intermittent (but not chronic) musculoskeletal symptoms around the time of HUNT2 may have affected insomnia prevalence at HUNT2. Potentially, also intermittent musculoskeletal symptoms between HUNT2 and HUNT3 may affect prevalence of CMSC at HUNT3, e.g. if caused by a severe injury leading to chronicity. Similarly, due to the lack of reliable and complete data, we were not able to adjust for medication use that may affect insomnia and/or pain. Furthermore, adjustment on rheumatic diseases like rheumatoid arthritis, ankylosing spondylitis and psoriasis-arthritis would have been preferable. However, self-reports of such diseases are often false-positive, and their prevalence is low [49]. Thus, adjustment was in this case unlikely to improve the model. Finally, residual confounding due to poorly measured or unmeasured factors cannot be ruled out. To reduce the possibility of selection bias, individuals with missing answers were included in the analyses. However, we are aware that this strategy also could bias the results due to residual confounding.

## Conclusion

In this large prospective population-based cohort study, insomnia was associated with increased risk of CMSC, CWMSC, and CMSC located in 3–4 or more body regions. The current results may indicate that prevention of insomnia could be an important target to reduce occurrence of CMSC and CWMSC.

## Abbreviations

ACR: American College of Rheumatology
BMI: Body mass index
CI: Confidence interval
CMSC: Chronic musculoskeletal complaints
CNS: Central nervous system
CWMSC: Chronic widespread musculoskeletal complaints
DSM (-IV and -V): Diagnostic and statistical manual of mental disorders
FM: Fibromyalgia
HADS: Hospital anxiety and depression score
HUNT: The Nord-Trøndelag Health Study
ICD-10: International classification of diseases 10
ICSD-3: International classification of sleep disorders 3
NTNU: Norwegian University of Science and Technology
REK: Regional Committee for Ethics in Medical Research
RR: Risk ratio
WPI: Widespread pain index
